# Monthly Summary

**Published:** 1876-12

**Authors:** 


### MONTHLY SUMMARY.

*Differences between the Anæsthesia of Ether and Chloroform*—Prof. Schiff, relates the results of more than five thousand experiments on the differences between ether and chloroform anæsthesia. With both paralysis of conscious sensation, paralysis of the movement of voluntary muscles, paralysis of respiration, circulation, and, finally, paralysis of the heart and the vaso-mortor nerves occurs. Respiratory paralysis is produced by ether when circulation and blood pressure remains within the limits compatible with life Sometimes the vascular pressure increases, sometimes it decreases, but is always sufficiently high to allow the exchange of the carbonic acid gas with the oxygen of the atmosphere. Vascular succeeds respiratory paralysis when ether is administered. The reverse takes place with chloroform. Frequently an amount of this anæsthetic agent which would not be sufficient to produce respiratory may suffice to bring on vascular paralysis. Under these conditions, and when vascular paralysis lasts under thirty seconds, artificial respiration is useless, because there is no longer any exchange of gases, the blood pressure being diminished. The cessation of respiration, therefore, is not the most dangerous moment to animal life, when etherization is employed, whilst it may be so with chloroformization, because sometimes it is possible to produce some automatic respiratory movements, but nevertheless, respiration ceases immediately and the animal dies. With etherization, on the contrary, when some automatic inspirations are obtained, it may be taken as certain that re spiration will continue. and that the animal will live. Schiff affirms that in the present state of our science there are no means which will show us how to recognize, so as to prevent them, the tendencies which may cause death in some animals after the first inhalations of chloroform before having produced true anæsthesia. The reverse occurs with ether, so that it may be said that, in the present state of knowledge, the surgeon is responsible for the death of the individual by etherization, whilst he is not responsible when death occurs from chloroformization. From these facts Schiff concludes :

1. The phenomena relating to the paralysis of sensibility and movement are common to both ether and chloroform.

2. The two other orders of phenomena—that is to say, those relating to vascular and respiratory paralysis—often show themselves in inverse order with reference to these two agents.

3. With chloroform, however, either the one or the other of these two paralyses may first show itself, involving great danger to the animal if the vascular phenomena be the first to make their appearance. Therefore the use of chloroform should be rejected and ether only used.—*British Medical Journal.*

---

*Physiology of the Nerve Centres.*—The following are Dr. Brown-Sequard's recently promulgated views:—

1. As regards localization of function, a great many facts lead to the view that the nerve-cells endowed with the same function, instead of forming a cluster, so as to be in the neighborhood of each other, are scattered in the brain, so that any part of that organ can be distroyed without the cessation of their function. It makes no difference whatever whether the distance between nerve-cells employed in the same function is a small fraction of a millimetre or very much greater, as in either case their communications with each other must take place by conductors (nerve-fibres,) the length of which is unable to interfere with the function.

2. Each half of the brain is a complete brain originally, and possesses the aptitude to be developed as a centre for the two sides of the body, in volitional movement, as well as in all the other cerebral functions. Still, very few people develop very much, and perhaps nobody quite fully, the powers of the two brains; and, on the contrary, in most persons only one of these two primitively similar organs acquires great power for certain actions, and the other for other actions.

3. Communications between the body and the brain can be more or less fully accomplished by means of a very much smaller number of conductors than would be necessary according to any view like the well-known clavier theory. As we know that the will gives an order, and as we know by clinical facts that any part of the medulla oblongata can be destroyed without paralysis and that in some cases a very small portion of it has proved sufficient for the persistence of voluntary movements, it would seem that the order may be transmitted as well by one fibre as another, and that it is necessary to recognize the existence of faculties of a much higher order in the nerve cells of the spinal cord than those which are admitted to exist there. Many facts and a similar reasoning tend also to show that the nerve-cells of the spinal cord possess, as regards sensibility, faculties of a higher order than those which are admitted.—*Med. and Surg. Reporter.*

*Headache from Eye-Strain.*—Dr. Weir Mitchell, in the *American Journal of Medical Science,* observes that few but ophthalmic surgeons are aware of the need of interrogating the eye for answers to some of the hard questions which are put to us by certain head-symptoms; and he wishes to impress upon the profession—1st. That there are many headaches which are due indirectly to disorders of the refractive or accommodative apparatus of the eyes. 2d. That in these instances the brain symptom is often the most, and sometimes the sole, prominent symptom of the eye-trouble, so that while there may be no pain or sense of fatigue in the eye, the strain with which it is used may be interpreted solely by occipital or frontal headache. 3d. That the long continuance of eye-trouble may be the unsuspected source of insomnia, vertigo, nausea, and general failure of health. 4th. That in many cases the eye-trouble becomes suddenly mischievous, owing to some failure of the general health, or to increased sensitiveness of the brain from moral or mental causes.—*Journal of Materia Medica.*

---

*Obliteration of Depressed Cicatrices.*—Operation for, after Glandular Abscesses of Exfoliation of Bone. Mr. William Adams has performed this operation with great success. So many faces are rendered unsightly by these deep cicatrices, that any operation which results in the removal of the deformities must be a blessing to those afflicted by them. The operation is original, and consists in subcutaneously dividing all the deep adhesions of the cicatrix, by a tenotomy-knife introduced a little beyond the margin of the cicatrix, and carried down to its base, so as to carefully and thoroughly evert the cicatrix which remains prominently raised. Two hare-lip pins or fine needles are then passed through the base, at right angles to each other, so as to maintain the cicatrix in its everted and raised position, where it is so retained for three days. At the end of this time the needles are removed, and the somewhat swollen and infiltrated cicatricial tissue is allowed to settle gradually down to the proper level of the skin. He gives the full history of three such operations in his paper. The permanency of the cure is illustrated by cuts of two cases, in one of which the operation was done nine, and in the other three years ago. The depressions seem to be completely obliterated—*British Medical Journal.*

---

*Advance in Dental Surgery.*—More than a year since Dr. Geo. H. Perine of N. Y. City, introduced to the dental profession the Galvanic Cautery Battery, strongly recommending its employment in oral surgery. The advantages set forth are that the operation is instantaneous, painless, without shock and without hemorrhage, which is of great importance in operations in the mouth. So delicate are such operations, and particularly difficult is it to control the hemorrhage from vessels that cannot be conveniently ligatured, the cautery becomes invaluable. Dr. Perine recommends this instrument also for treating alveolar abscess, hemorrhage after extracting teeth, cauterizing nerves of the teeth when exposed, also for obtunding sensitive dentine, the instrument may be applied he says with the most happy effect, and thus facilitate the after operation.

Dr. Perine performed for a lady a few days since a very delicate and successful operation with cautery, removing a large tumour from the mouth, which he performed in a very short space of time, much to the gratification of his patient, also the professional friends who witnessed the operation. By the means that the doctor has introduced, and with his improved instrument the practice of oral surgery becomes emendated, and is shorn of very much of its terrors and danger.—*Medical News.*

---

*Ulceration of the Frenum Linguæ in Whooping Cough.*—At a meeting of the Harveian Society in London, Dr. Morton called attention to the frequency of this symptom. This coincidence of ulceration in this particular position with pertussis is not new, though English authors have not referred to it, except casually, in association with stomatitis. This ulceration has been described in both French and German literature, more especially by Bouchut, in his works on diseases of children and new-born infants; though what relationship it has to pertussis, or why it exists at all in that position, is not decided. It is probably owing to irritating secretions.—*Medical and Surgical Reporter.*

---

*Inflammatory Process—Its Nature.*—Dr. Thin (*Edinburgh Med Journal*, March,-April, 1876) in a series of articles makes the following points respecting the inflammatory process:

1. The changes in an inflamed tissue are divisible into two categories, the *destructive* and the *reparative.*

2. The destructive changes consist, as regards the fibrillary tissue, in swelling, softening, chemical change and disintegration; as regards the cellular elements, in division of the nuclei into small parts, swelling, death and disintegration of the cell. The destructive change extends towards the blood vessels or directly implicates them; the weakened vessels permits the escape of the fluid and formed elements of the blood.

3. When the cause of primary destruction is removed or exhausted, the process of repair begins by organization of the plasma and colorless blood cells. From the former the ground substance (fibrillary tissue, etc.) is formed ; from the latter all the cellular elements of what form sores. Flat cells, spindle and stellate cells, non striped muscle cells, epithelial cells, all owe their origin to the lymph or colorless blood corpuscles. But the type of cell once established by the development of the original corpuscle, there is afterwards neither retrogression nor metamorphosis. With the development of the free lymph cell of the fluids into a fixed element of the tissue the power of reproduction is lost.—*Detroit Review of Medicine & Surgery.*

---

*Composition of the Human Body.*—A complete analysis of man recently made by Dr. Lancaster, of London, has been described by him in a chemical lecture. The body operated upon weighed 158.4 lbs., and the lecturer exhibited on the platform 23.1 lbs. carbon, 2.2 lbs. lime, 22.3 ounces phosphorus, and about 1 ounce each of sodium, iron, potassium, magnesium, and silicon. Dr. Lancaster apologized for not exhibiting 5,585 cubic feet of oxygen, weighing 121 lbs., 105,000 cubic feet of hydrogen, weighing 15.4 lbs., and 52 cubic feet of nitrogen likewise obtained from the body, on account of their great bulk. All of these elements combine into the following: 121 lbs., water, 16.5 lbs., gelatine, 52 lbs. fat, 8.8 lbs. fibrin and albumen, 7.7 lbs. phosphate of lime and other mineral substances.—*Am. Gaslight Journal.*

---

*Cement for Rubber and Glass.*—A good cement, that will render India-rubber in any form adherent to glass or metal, is oft-times a desideratum, and in the *Polytechnisches Journal* for March the following simple recipe is given for the preparation of such a compound : Some shellac is pulverized, and then softened in ten times its weight of strong ammonia, whereby a transparent mass is obtained, which becomes fluid after keeping some little time, without the use of hot water. In three or four weeks the mixture is perfectly liquid, and, when applied, it will be found to soften the rubber. The rubber hardens as soon as the ammonia hes evaporated, and thus becomes impervious to both gases and liquids. For cementing rubber material in any shape, to metal, glass, or any such surfaces, this cement is strongly recommended.

---

*Influence of the Age of the Mother on the Sex of the Child.*—In the Obstetricle Hospital of Munich, between June 1, 1859, and December 31, 1875, there were 13,469 cases of labor, with 13,621

births Of these children, 7,021 were males, and 6,600 were females, the proportion being 106.4 : 100. Dr. Hecker, who classified these cases, found that 432 primiparæ, who had passed their 30th year, were delivered of 247 male and 185 female children, the proportion being 133 : 100. The doctor thinks that the age of the mothers had some influence in deciding the sex of the children. Other observers had previously obtained similar results. Thus Ahlfed found that of 102 children whose mothers were primiparæ over 30 years of age, 50 were boys and 43 girls proportion of 137 : 100. Schramm collated the cases of 599 primiparæ over 30 years of age, who were delivered of 348 boys and 262 girls, being a proportion of 132.8 : 100. In the cases classified by Dr. Hecker, the age of the father did not seem to exert any influence on the sex of the child. Ahlfeld thought that there are male and female ova in the ovaries, but if so, it would be difficult to account for the fact that more males than female ova are matured in women who do not become pregnant until they have reached 30 years of age.

---

*Death from Ether.*—Professor E. L. Holmes, of Chicago, gives the particulars of a case of this unusual accident occuring in his practice. The patient was a male, age seventy-four years, and very obese ; height four feet eight inches, weight one hundred and eighty four pounds ; general health good ; slightly asthematic. He had cataract, and in the latter part of December, '75, preliminary iridectomy was performed under ether. Unpleasant symptoms were observed, but the patient recovered from the anæsthetic and from the operation without any bad results. On March 27th, '76, ether was again administered ; after about half a pound of Squibb s stronger ether had been given, violent coughing set in, soon followed by extreme lividity of the face and complete cessation of breathing. Under proper treatment respiration was reestablished and the operation of extraction of the lens was rapidly performed. This did not occupy more than two minutes, no more ether having been administered. Breathing was then observed to have ceased, the face was very livid and the pulse weak. In a minute more the heart had ceased acting, the patient was dead. No autopsy was obtained. —*Chicago Med. Jour. and Examiner.*

---

*Sulphide of Calcium.*—Dr. T. Curtis Smith (*Southern Med. Record*, July '76) confirms the observations of Ringer upon the value of this agent in boils, abscesses, carbuncles, and glandular enlargements. There is no other remedy which will cause the resolution of these affections equally well. He gives it in powder, with sugar, or in pill form, in the dose of one-half grain, every four or six hours, for children. Adults may take from one half to two grains, with sugar, four or six times a day.

---

*Mercurial Poisoning from Canned Meat.*—At a recent meeting of the Chemical section of the New York Academy of Sciences, Prof. Folke stated that, on opening a can of cooked corn beef, put up by a company in Chicago, he noticed some bright metallic globules, which proved to be metallic mercury. Beside these, a considerable quantity of combined mercnry was present in the form of albuminate of mercury. How the poison came in the meat is a mystery; but a member suggested that, inasmuch as thermometers are employed to regulate the temperature when canning, the mercury may have come from a broken thermometer. It may be interesting to note that a case of poisoning has been reported in Boston from eating canned cooked corned beef. Another member of the Academy stated that he too, had suffered severely after eating two ounces, but whether from mercury he could not say.—*Med. and Surg. Reporter.*

---

*Poisonous Properties of Glycerine.*—M. Dujardin Beaumetz, havsng noficed the tendency to employ glycerine internally, in the treatment of diabetes for instance, and believing that it ought to be researved for external use, was induced to investigate its effecss on the economy when administered in large doses. Berthelot has shown that this shbstance is a triatomic alchhol, and it should consequently produce effects idəntical with those of alcohol. The following are the conclusiods he has derived from his experiments:

1. When chemically pure glycerine is introduced under the skin of a dcg in quantities of from 2 to 4½ drachms for every 2½ pounds of the weight of the body, it causes death within twenty-four hours.

2, Symptoms of acute glycerine resemble, within certain limits, those of acute alcoholism.

3. The necroscopic lesions of acute glycerism are analogous to those of alcoholism, which leads to the supposition that the toxic actions of the two substances are very nearly the same.

4. From a therapeutic standpoint, the introduction of large quantities of glycerine into the body io not devoid of danger.

It is necessary then, to use care in prescribing glyeerine for internal use, and not to lose siget of the fact that, like alcohol, glycerine will prove a successful or an unsuccessful remedy according to its dose and mode of administration.—*La Foance Medicale*'

